# Inflammation Control and Tumor Growth Inhibition of Ovarian Cancer by Targeting Adhesion Molecules of E-Selectin

**DOI:** 10.3390/cancers15072136

**Published:** 2023-04-04

**Authors:** Bowen Yang, Shanmei Yin, Zishuo Zhou, Luyao Huang, Mingrong Xi

**Affiliations:** 1 Department of Gynecology and Obstetrics, West China Second University Hospital, Sichuan University, Chengdu 610041, China; 2 Key Laboratory of Birth Defects and Related Diseases of Women and Children (Sichuan University), Ministry of Education, Chengdu 610041, China; 3 Key Laboratory of Drug-Targeting and Drug Delivery System of the Education Ministry and Sichuan Province, Sichuan Engineering Laboratory for Plant-Sourced Drug and Sichuan Research Center for Drug Precision Industrial Technology, West China School of Pharmacy, Sichuan University, Chengdu 610041, China

**Keywords:** ovarian cancer, E-selectin, bovine serum albumin

## Abstract

**Simple Summary:**

The aim of our study is to use E-selectin-binding peptide (ESBP) to actively recognize E-selectin, so allowing a drug delivery system to actively recognize the cells and inhibit ovarian cancer cells by targeting adhesion molecules of E-selectin. A drug-loaded nanoparticle with a special structure was designed as the drug carrier. Based on the fact that tumors often occur together with inflammation, the drug could be delivered to and more concentrated in ovarian tumor sites as well as tumor-associated inflammatory cells by using this drug-delivery system targeting selectins. Through recognition and binding with E-selectin in ovarian cancer cells and utilizing the steric hindrance effect of macromolecules that nanoparticles possess, paclitaxel was delivered and released, thus inducing ovarian cancer cell apoptosis and cell migration and decreasing myeloid-derived suppressor cells related to drug-resistance in a mouse model. It also had higher drug efficacy, extended the survival of tumor-bearing mice and had relatively lower in vivo toxicity than paclitaxel treatment. All results in this study provided potent proof of its potential to be applied to clinical use.

**Abstract:**

Objective: The aim is to use E-selectin-binding peptide (ESBP) to actively recognize E-selectin, so allowing a drug delivery system to actively recognize the cells and inhibit the tumor growth of ovarian cancer by targeting adhesion molecules of E-selectin. An ovarian-cancer-directed drug delivery system was designed based on the high affinity of E-selectin-binding peptide (ESBP) to E-selectin. The effects and mechanisms of ESBP-bovine serum albumin (BSA) polymerized nanoparticles were investigated. Methods: BSA polymerized nanoparticles (BSANPs) and ESBP-BSANPs-paclitaxel (PTX) were prepared and their characteristics were measured. The in vitro targetability and cytotoxicity of ESBP-BSANPs-PTX were evaluated through in vitro drug uptake and MTT experiments. The mechanisms of ESBP-BSANPs-PTX were investigated via apoptosis, wound healing and immunohistochemistry assays. The in vivo targeting properties and drug effects were observed in a mouse tumor-bearing model. Results: In vitro experiments revealed an increase in the uptake of ESBP-BSANPs-FITC. The cytotoxicity of ESBP-BSANPs-PTX in A2780/CP70, HUVEC, RAW264.7 and ID8 cells was higher than that of PTX alone. ESBP-BSANPs-PTX increased cell apoptosis in a dose-dependent manner and exhibited a greater ability to inhibit cell migration than BSANPs-PTX. In vivo experiments demonstrated the targetability and good effects of ESBP-BSANPs. Conclusions: ESBP-BSANPs-PTX improve PTX targetability, provide tumor-specific and potent therapeutic activities, and show promise for the development of agents in preclinical epithelial ovarian cancer.

## 1. Introduction

Treating ovarian cancer (OC) with both anti-recurrence and anti-metastatic therapies, while maintaining high-efficiency and low-toxicity, is challenging. OC has the highest mortality rate among the malignant tumors of the female reproductive system, and most patients are in the advanced stage [1]. The combination of surgery and systemic chemotherapy is the standard treatment for OC [2,3,4,5]. However, first-line chemotherapy drugs [4] such as paclitaxel (PTX) and cisplatin cannot accurately target ovarian cancer cells, and also have relatively large systemic side effects [6,7,8,9]. This makes it difficult to distribute the drugs effectively to tumor sites, leading to low targeting efficiency and treatment difficulties [10,11].

At present, targeted drugs such as anti-angiogenic drugs, PARP inhibitors and immune checkpoint inhibitors are applied for clinical use [12,13]. These drugs increase the platinum-free interval and chemotherapy-free interval and improve patient prognosis [5]. National Comprehensive Cancer Network (NCCN) guidelines recommend their use based on various clinical trials. Those targeted therapies still have defects, such as systemic toxicity and side effects [14]. In addition, their high costs can also place significant financial burdens on patients, insurance companies and local medical systems.

Based on current research findings, reducing expenses, improving chemotherapeutic drug targetability, increasing drug concentrations at tumor sites and decreasing toxicity are very crucial.

The process through which malignant tumor cells adhere to vascular endothelial cells and migrate to other tissues is similar to the immune response in which leukocytes are recruited to the inflammatory site or site of injury [15,16,17,18]. The same adhesion molecules are involved, including selectins, integrins and their ligands. Selectins are transmembrane proteins, which mainly mediate cellular interactions by recognizing certain glycoproteins on cell membranes and binding to receptor ligands. Obviously, selectins have effects on inflammatory reactions and tumor metastasis [15]. E-selectin (CD62E) is constitutively expressed on vascular endothelium [19] and is upregulated in microvasculature when a tumor presents. E-selectin is highly expressed in ovarian cancer tissues, and E-selectin-binding peptide (ESBP) can actively recognize and bind to E-selectin. Since inflammation occurs in tumor sites, directing or delivering drugs to the tumor sites by recognizing and targeting the selectins expressed by inflammatory cells can be promising.

Nanoparticles have been widely used as drug delivery systems [20]. Nab-paclitaxel (NPT), nanoparticle albumin-bound paclitaxel, was approved by the FDA in 2005, and addresses the hydrophobicity problem of PTX, thereby reducing the need for organic solvents and the occurrences of allergic reactions in patients [21]. However, nab-paclitaxel still cannot target cancer cells. Due to the enhanced permeation and retention (EPR) effect [22], also known as passive targeting, the particle size of NPT allows PTX to be partly delivered to and gathered in target sites, but most PTX is distributed to the liver and endothelial system and then inactivated.

In this study, a drug-loaded nanoparticle with a special structure targeting E-selectin was designed. Using the steric hindrance effect of macromolecules in the nanoparticle, the drug was delivered to the target site through the binding of ESBP to the E-selectin, and a higher therapeutic concentration can be achieved in ovarian cancer cells, thus having a better inhibition in ovarian cancer cells.

## 2. Materials and Methods

### 2.1. Preparation and Characterization of E-Selectin-Binding Peptide Modified PTX-Loaded Bovine Serum Albumin Polymerized Nanoparticles (ESBP-BSANPs-PTX)

#### 2.1.1. Preparation of ESBP-BSANPs-PTX

Bovine serum albumin (BSA) (1.5%, *w*/*v*: 15 mg/mL) (Shanghai Bio Science & Technology Co., Ltd., Shanghai, China, 171205) aqueous solution was prepared. PTX (J&K Scientific Ltd., Guangzhou, China, CAS:33069-62-4) was dissolved in ethanol. The PTX solution was added to the BSA solution slowly, and then incubated for 1 h.

A 4-fold volume of ethanol was added dropwise to the BSA solution at a rate of 1.5 mL/min. Pentalhyde (0.5%, *w*/*w*) was added for cross-linking solidification for 12 h. The current solution was subjected to rotary evaporation in a water bath at 40 °C to remove the ethanol. In a dark environment, the resulting pellet (PTX-loaded BSA nanoparticles [BSANPs-PTX]) was collected via high-speed centrifuge (10,000 rpm/min, 20 min), reconstituted, and then filtered 2 times through a 1.2 μm membrane.

ESBP (Qiangyao Biological Co., Ltd., Shanghai, China, 2019122005) (1 mg of ESBP dissolved in 50 µL of dimethyl sulfoxide [DMSO]) was added to the BSANPs-PTX solution and incubated at room temperature for 1 h. Succinimidyl iodoacetate (diluted in DMSO) was then added and the mixture was allowed to react at room temperature overnight to obtain ESBP-BSANPs-PTX.

Paclitaxel content was determined via high-performance liquid chromatography (HPLC). Twenty microliters of samples were separated at the following chromatographic conditions: DIKMA C18 column (250 mm × 4.6 mm, 5 μm) with a mobile phase of methanol/acetonitrile/water = 35/35/30 at a flow rate of 1.0 mL/min and with a detection wavelength of 227 nm at 30 °C. 

The paclitaxel was precisely weighed and dissolved in acetonitrile to prepare a stock solution. The stock solution was diluted with acetonitrile to concentrations of 1, 10, 20, 30, 40, 60 and 80 μg/mL, filtered with a 0.22 μm membrane, and loaded onto the column for analysis.

#### 2.1.2. Characterization of Nanoparticle Size, Potential, Morphology, Stability, Encapsulation Efficiency and Drug Load

The particle size, zeta potential and polydispersity index (PDI) of the sample were measured using dynamic light scattering (Zetasizer Nano ZS90, Malvern Instruments, Malvern, UK) at different time points in triplicate.

The encapsulation efficiency (EE) and drug load (DL) were determined using an ultrafiltration tube (Millipore, Burlington, MA, USA) with a cutoff of 10 kDa. Unencapsulated PTX was quantified using HPLC. The encapsulation efficiency was determined using the following formula:

EE (%) = (W_total_ − W_free_)/W_total_ × 100%

DL (%) = (W_total_ − W_free_)/W_NP_ × 100%

W_total_ is the total PTX content in the preparation, W_free_ is the unencapsulated free PTX content, and W_NP_ is the total dosage of the nanoparticles.

#### 2.1.3. Morphology

Transmission electron microscopy (TEM) (TEM HT7800, Hitachi, Ltd., Tokyo, Japan) was used to observe the morphology of the ESBP-BSANPs-PTX and BSANPs-PTX. The sample was stained with phosphotungstic acid, pH 6.5 (Beijing Zhongjingkeyi Technology Co., Ltd., Beijing, China), and then observed and photographed under TEM.

### 2.2. Immunohistochemistry (IHC) and Haematoxylin and Eosin (H&E) Staining

Paraffin sections of ovarian cancer, non-cancer ovarian tissues, and cells of ascites were used for immunohistochemical (IHC) examination with E-seclectin antibody (1:200, Proteintech, Wuhan, China, 20894-1-AP), Caspase 3 polyclonal antibody (1:200, Proteintech, 19677-1-AP), and anti-VEGFA antibody (1:200, Boster Biological Technology, Pleasanton, CA, USA, BA0407) as the primary antibodies. The horseradish peroxidase secondary antibody in a Dako REALTM EnVision^TM^ kit was used to visualize the antigen–antibody interaction. The sections were evaluated by two blinded pathologists, and the fraction score was defined as described below.

The expression of protein was assessed semiquantitatively according to criteria that accounted for both the fraction and intensity of immunostaining in the tumor cells involved. We used 10 randomly selected light microscope fields of a total of 1000 cells for each section. The fraction score was defined as follows: 0, negative, no tumor cell stained; 1, ≤10% of cells stained in the same type of cells; 2, 10% to 50% of cells stained; 3, >50% but ≤75% of cells stained; and 4, >75% of cells stained. The intensity score was defined as follows: 0, no appreciable staining in the tumor cells; 1, light yellow color, barely detectable staining in the cytoplasm and/or nucleus compared to the stromal elements; 2, readily appreciable brown staining; and 3, dark brown staining in tumor cells obscuring the cytoplasm and/or nucleus. The total score was calculated by multiplying the fraction and intensity score. For statistical analysis, total scores of 0–3 were considered negative (−); 3 < total scores ≤ 7 were (+); 7 < total scores ≤ 9 were (++), 9 < total scores ≤ 12 were (+++). Total scores ≥6 were considered as high expression and total scores <6 were low expression.

The sections were counterstained with haematoxylin for detection. The obtained tissues were fixed in 4% paraformaldehyde overnight. Afterwards, they were dehydrated in 25% sucrose, sectioned into 4 μm slices and stained with H&E. The stained sections were imaged under an inverted phase-contrast microscope (Olympus, Tokyo, Japan, BX53).

### 2.3. Cell Culture

Six cell lines were used in this study: human ovarian cancer cell lines SK-OV-3, A2780/CP70; human umbilical vein endothelial cell line (HUVEC); mouse ovarian cancer cell line ID8; mouse leukemia cell line RAW264.7 and human granulosa-like tumor cell line KGN. RAW264.7 and KGN cell lines were used as controls. 

SK-OV-3 cells were kindly provided by the Stem Cell Bank, Chinese Academy of Sciences. A2780/CP70, HUVEC, RAW264.7 and ID8 cells were obtained from the Key Laboratory of Gynecological Oncology, West China Second University Hospital, Sichuan University. The human granulosa-like tumor cell line KGN was from the Reproductive Endocrinology Laboratory, West China Second University Hospital, Sichuan University.

SK-OV-3 and ID8 cells were cultured in DMEM; A2780/CP70 in RPMI; KGN in DMEM/F12; and HUVEC in M199/EBSS media. All cell lines were supplemented with 10% fetal bovine serum (FBS), 100 U/mL penicillin and 100 µg/mL streptomycin, and maintained in a humidified incubator with 5% CO_2_ at 37 °C. Each cell line was passaged every 4–6 days. All assays were performed in their respective cell media.

### 2.4. Determination of Cell Uptake

ESBP-BSANPs-FITC and BSANPs-FITC were prepared to observe the cellular drug uptake of fluorescein isothiocyanate (FITC) (simulating PTX) in HUVEC, SK-OV-3, A2780/CP70, ID8 and RAW264.7 cells from drug-loaded ESBP-BSANPs. Cell lines KGN and RAW264.7 were used for comparisons. Dilutions of ESBP-BSANPs-FITC, BSANPs-FITC or free FITC (Biofroxx, Einhausen, Germany, 2284MG050) were added to cells at a final FITC concentration of 100 ng/mL and incubated with the cells for 2 h. The uptake results were compared using fluorescence intensity quantified by flow cytometry with a free FITC control. The normalized uptake results for each cell line were presented by the ratio, which is the fluorescence intensity of ESBP-BSANPs-FITC and BSANPs-FITC group cells divided by the fluorescence intensity of the free FITC control group cells of each cell line. This ratio indicates an increase in the relative fluorescence intensity. 

### 2.5. Cytotoxicity

The in vitro cytotoxic effects of free PTX, ESBP-BSANPs-PTX, BSANPs-PTX and BSANPs on SK-OV-3, A2780/CP70, ID8, KGN and RAW264.7 cells were examined and compared at a range of relative PTX concentrations using the MTT (3-[4,5-dimethylthiazol-2-yl]-2,5 diphenyl tetrazolium bromide) assay. Cultured cells were seeded into 96-well plates (4000 cells/well) and were cultured for 24 h. Cell culture media were used for drug dilution and PBS was used in the preparation of a stock solution, so the 0 (vehicle) treatment group was set as the blank (medium only) group, and its cell viability was considered as the baseline 100%.

After 24 h, 48 h and 72 h of incubation, medium containing the drug in each well was aspirated, 20 µL 5 mg/mL MTT (Biofroxx, 1334MG250) solution and 80 µL of fresh media were added and cells were incubated for another 4 h. The liquid in each well was replaced by 150 µL of DMSO, and then the absorbance of each well at a wavelength of 490 nm was measured using a microplate reader (Varioskan LUX, Thermo Fisher Scientific, Waltham, MA, USA). The cell viability rate was calculated according to the following formula, and the median inhibitory concentration (IC50) was estimated based on the survival curve.

Viability = ([Abs]_sample_ − [Abs]_blank_)/([Abs]_control_ − [Abs]_blank_) × 100%

### 2.6. In Vitro Annexin V/PI Apoptosis Detection

The effects of ESBP-BSANPs-PTX on ovarian cancer cell apoptosis were measured using flow cytometry. SK-OV-3 and A2780/CP70 cells were separately cultured in media with free PTX, ESBP-BSANPs-PTX, BSANPs-PTX and BSANPs at 37 °C. After 72 h of culture, cells were collected, centrifugated, washed twice with PBS, prepared as suspension, stained using an Annexin V/PI Apoptosis Kit (DOJINDO, AD10), and subjected to flow cytometry analysis within 1 h according to the manufacturer’s protocol.

The apoptotic cells were calculated as early apoptotic cells (PI-/Annexin V+); the sum of apoptotic and necrotic cells was calculated as Annexin V+ cells (PI-/Annexin V+ and PI+/Annexin V+); the live cells were PI-/Annexin V−. The solvent of ESBP-BSANPs-PTX and BSANPs-PTX was PBS, and the solvent of free PTX contained very little DMSO; SK-OV-3 and A2780/CP70 cells cultured in complete medium were used as the control groups.

### 2.7. Wound Healing Assay

The effects of free PTX, ESBP-BSANPs-PTX, BSANPs-PTX and BSANPs on cell migration were observed and evaluated using a wound healing assay by measuring the wound width at different time points. 

SK-OV-3 and A2780/CP70 cells were seeded in 6-well plates, and the cell monolayers were scratched with a 200 µL pipette tip after the cells had attached to the bottom. After scratching, cells were cultured with serial dilutions of the drug with 1% FBS medium. The wounds were observed and imaged using an inverted phase-contrast microscope (4×, bright field) with a 200 µm scale bar.

### 2.8. In Vivo Targeting Examination

The mouse subcutaneous tumor model was obtained by subcutaneously injecting 5 × 10^6^ ID8 cells in 100 µL of Matrigel (1:9 dilution with PBS) into the shaved right flank of each mouse and using this for in vivo imaging experiments. The tumor-bearing mice were randomly divided into 4 groups: (1) free 1,1′-dioctadecyl-3,3,3′,3′-tetramethylindodicarbocyanine perchlorate (DID), (2) ESBP-BSANPs-DID, (3) BSANPs-DID, and (4) blank control.

Free DID (1,1′-dioctadecyl-3,3,3′,3′-tetramethylindodicarbocyanine perchlorate), ESBP-BSANPs-DID and BSANPs-DID were injected intravenously in the tail into mice at a 2 μg/mouse equivalent DID dose. After 0, 2, 6, 12 and 24 h of administration, fluorescence imaging of the mice was performed using photoacoustic imaging (Caliper, Hopkinton, MA, USA). After imaging, the mice were sacrificed, and their hearts, livers, spleens, lungs and kidneys were collected, fixed in paraformaldehyde, and subjected to fluorescence intensity determination using the same machine. Untreated mice were used as blank controls. This study was approved by the Ethics Committee of West China Second University Hospital, Sichuan University.

### 2.9. In Vivo Antitumor Assessment

The in vivo antitumor effects of ESBP-BSANPs-PTX were evaluated using tumor-bearing mice. The 6–8-week-old female C57BL/6 mice (Chengdu Dossy Experimental Animal Co., Ltd., Chengdu, China) were intraperitoneally injected with 5 × 10^6^ ID8 cells in 100 µL of Matrigel (1:9 dilution with PBS). 

The mice were randomly divided into 4 treatment groups: (1) ESBP-BSANPs-PTX group, (2) BSANPs-PTX group, (3) free PTX group, (4) vehicle-treated control group. The drug for each group was administered at a dose of 10 mg/kg body weight via tail vein injection once every week for 4 consecutive administrations. The mice were monitored each day, and the body weights were measured daily.

H&E staining was used to observe pathological changes in the organs (heart, liver, spleen, lung, kidney) and tumor nodules (methods described in Section 2.2).

The sections were examined and evaluated by two pathologists and compared with those of normal unmodeled mouse organs for the possible presence of pathological changes and whether the subcutaneous tumor model was successfully established. 

#### 2.9.1. In Vivo Toxicity Test

The mice were monitored the day after administration, and the time of death or symptom onset was recorded. Daily observations were continued for 48 days after the first drug administration to track lethality and measure the weights of surviving mice. Observation continued for 48 days after the 1st drug administration in order to determine if there was any toxicity. On the 48th day, the mice were then sacrificed and whole blood was collected from the mouse orbit. For the measurement of hematological parameters, an Auto Hematology Analyzer (Shenzhen Mindray Animal Medical Technology Co., Ltd., Shenzhen, China, BC-2800 Vet) and Cobas^®^ 6000 analyzer series (Roche Diagnostics International AG, Rotkreuz, Switzerland) were used.

The heart, liver, spleen, lungs and kidneys were dissected and weighed. H&E staining (Methods described in Section 2.2) was used to observe the pathological changes. All operation procedures were in accordance with the requirements of the ethical approval.

#### 2.9.2. In Vivo Drug Effects

To investigate the in vivo drug effects, flow cytometry was used to analyze the apoptosis changes and detect myeloid-derived suppressor cells (MDSCs) in the abdomen. On the 48th day after the 1st drug administration, the mice were then sacrificed, ascites or peritoneal washing fluid were collected, and the pellet was separated via centrifuging. An Annexin V/PI Apoptosis Kit was used to analyze apoptosis changes and compare differences in each treatment group. PE anti-mouse CD45 antibody (BioLegend, San Diego, CA, USA, 103,106/200 µg), APC anti-mouse Ly-6G/Ly-6C (Gr-1) antibody (BioLegend, 108,412/100 µg) and FITC anti-mouse/human CD11b antibody (BioLegend, 101,206/500 µg) were used as antibodies to detect MDSCs. Then, samples were subjected to flow cytometry analysis under the guidance of the manufacturer’s protocol.

Ascites, peritoneal washing fluid and tumor tissues from different treatment groups were stained using Caspase 3 Polyclonal antibody and Anti-VEGFA antibody to detect caspase 3 and VEGFA via IHC (methods described in Section 2.2).

#### 2.9.3. Survival Curve

The survival time for each treatment group was recorded, and the end time point was 120 days. Mice were euthanized individually when moribund or as a cohort after 120 days.

### 2.10. Statistical Analysis

The results are presented as mean ± SD. Statistical significance for comparisons were assessed using the 2-tailed Student’s *t*-test when comparing two groups and using one-way ANOVA and Dunnett’s multiple comparisons when comparing multiple groups. A *p* < 0.05 was considered statistically significant. In figures, ns represents *p* > 0.05; * *p* < 0.05, ** *p* < 0.01, *** *p* < 0.001 and **** *p* < 0.0001.

## 3. Results

### 3.1. Preparation and Characterization of ESBP-BSANPs-PTX

#### 3.1.1. Characterization of ESBP-BSANPs-PTX

**Figure 1:**
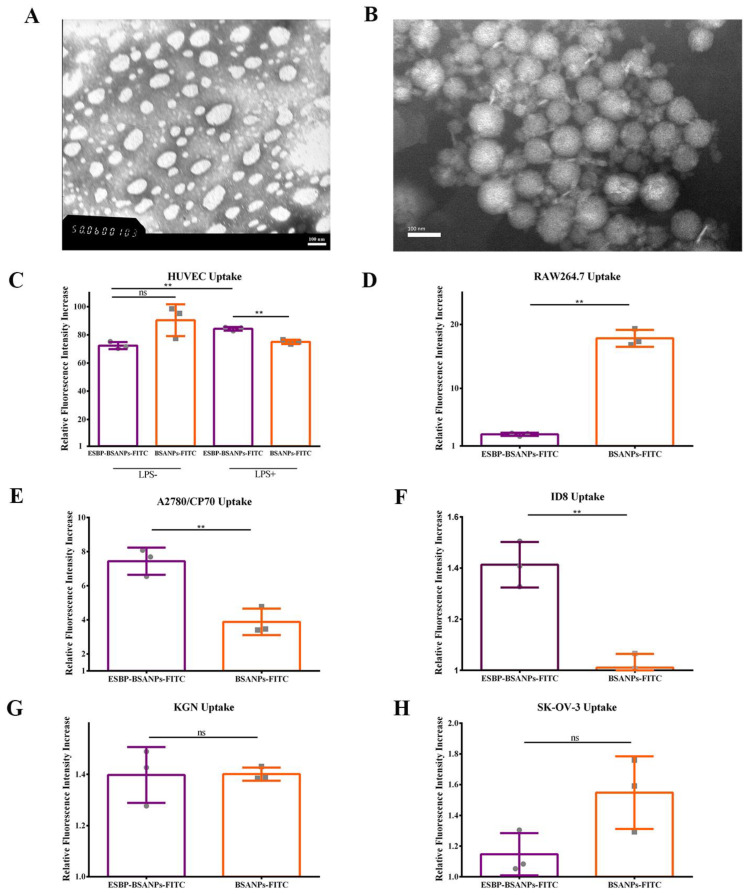
Preparation and characterization of ESBP-BSANPs-PTX. Transmission electron microscope (TEM) images of (**A**) BSANPs-PTX and (**B**) ESBP-BSANPs-PTX (scale bar: 100 nm). The normalized uptake of each cell line (uptake of ESBP-BSANPs-FITC and BSANPs-FITC groups compared with that of free FITC group) (mean ± SD, *n* = 3). (**C**) HUVEC and (**D**) RAW264.7 cells are E-selectin expressing cells; (**E**) A2780/CP70 and (**F**) ID8 are CD44 low-expression cells, (**G**) KGN and (**H**) SK-OV-3 are CD44 high-expression cells. ns represents *p* > 0.05, ** *p* < 0.01.

ESBP-BSANPs-PTX and BSANPs-PTX had a spherical shape with a smooth surface (Figure 1A,B). ESBP-BSANPs-PTX were normally distributed, with a diameter of 172.57 ± 14.63 nm, PDI of 0.193 ± 0.073 and zeta potential of −59.7 mV; BSANPs-PTX had a Z-average size of 150.17 ± 25.27 d.nm, PDI of 0.178 ± 0.087 and zeta potential of −59.7 mV. BSANPs had a Z-Average size of 155.4 ± 31.07 d.nm, PDI of 0.13 ± 0.038 and zeta potential of −16.5 mV.

The encapsulation rates of ESBP-BSANPs-PTX and PTX content were measured using HPLC. Within the PTX range of 10–70 µg/mL, a linear regression curve of y = 36642x + 18723 (y means peak area, x means PTX concentration [µg/mL]) was obtained with R^2^ = 0.9993 and this was used for subsequent PTX quantification and encapsulation efficiency determination. 

The results showed that the PTX encapsulation efficiency was 76.42 ± 3.67% for ESBP-BSANPs-PTX and 81.44 ± 9.09% for BSANPs-PTX, while the drug load was 2.14 ± 1.57% for ESBP-BSANPs-PTX and 2.29 ± 1.79% for BSANPs-PTX. 

#### 3.1.2. Successful In Vitro Drug-Delivery

ESBP-BSANPs demonstrated selective drug delivery. Remarkably, all cell lines had a higher uptake of ESBP-BSANPs-FITC and BSANPs-FITC compared to free FITC control (Figure 1C–H), with normalized uptake results higher than one, indicating successful drug delivery in these cell lines. HUVEC, A2780/CP70 and ID8 cells could uptake ESBP-BSANPs-FITC with a higher fluorescence intensity than BSANPs-FITC (Figure 1). HUVEC and RAW264.7 are E-selectin expressing cells, and E-selectin can be highly increased when inflammation happens. Lipopolysaccharide (LPS) can create an inflammatory environment and induce cell injury to increase the expression of E-selectin in HUVEC [23]. After HUVEC cells were activated via LPS, the uptake of ESBP-BSANPs-FITC was increased (*p* < 0.01) compared with cells without LPS activation, and it was significantly higher than that of BSANPs-FITC (*p* < 0.01). Without LPS activation, the uptake of ESBP-BSANPs-FITC by RAW264.7 cells was lower than that of BSANPs-FITC (*p* < 0.01).

The fluorescence intensity of the ESBP-BSANPs-FITC group was higher than that of the free FITC or BSANPs-FITC group in the cell lines with low CD44 expression, A2780/CP70 and ID8 (*p* < 0.01). In the cell lines with high CD44 expression, SK-OV-3 and KGN, the uptake results of ESBP-BSANPs-FITC were similar to BSANPs-FITC (*p* > 0.05).

### 3.2. Higher Cell Cytotoxicity of ESBP-BSANPs-PTX than Free PTX

**Figure 2:**
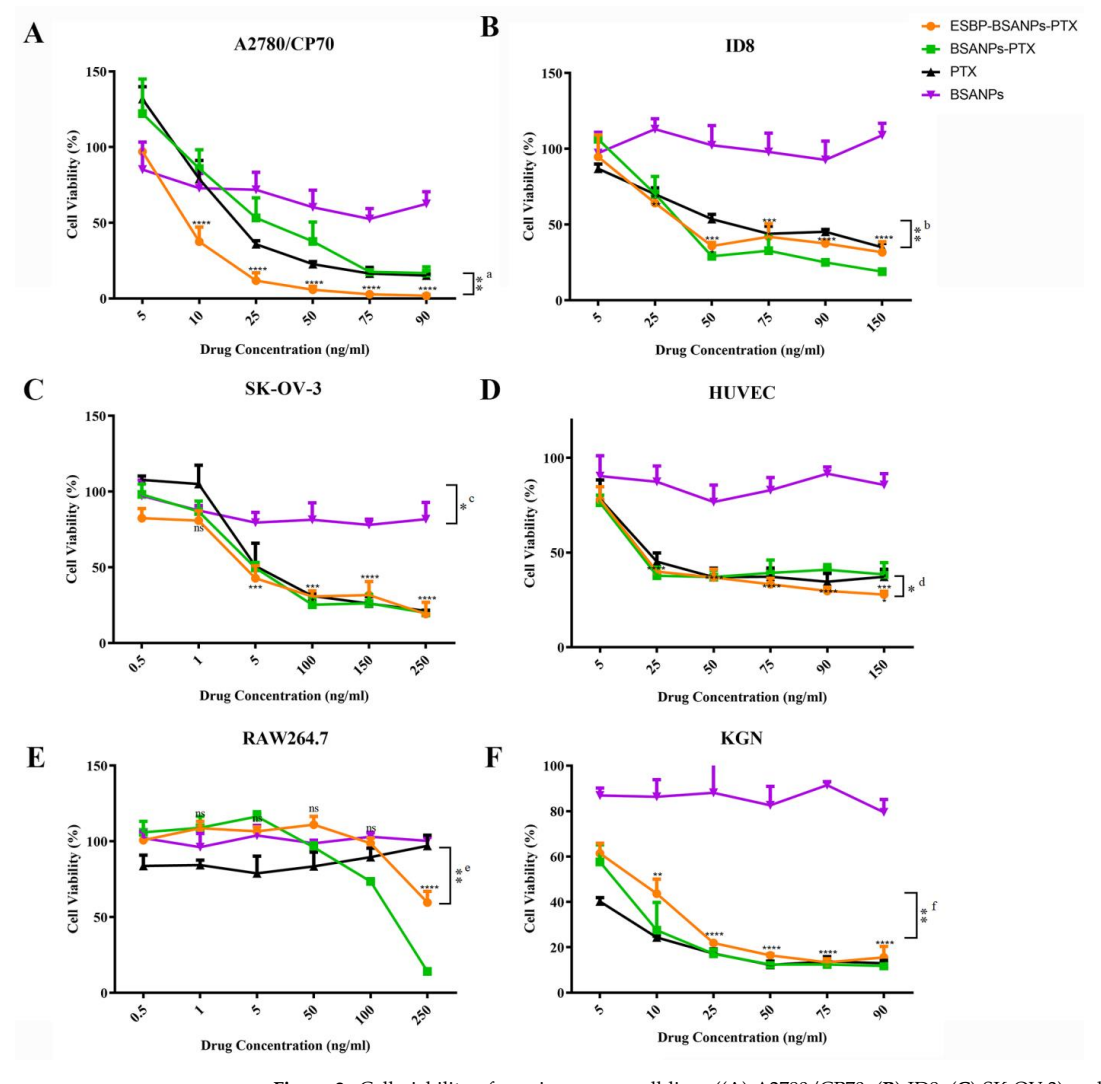
Cell viability of ovarian cancer cell lines ((**A**) A2780/CP70, (**B**) ID8, (**C**) SK-OV-3) and (**D**) HUVEC, (**E**) RAW264.7 and (**F**) KGN cells at 72 h time point was analyzed using microplate reader (mean ± SD, *n* = 3). Serial dilutions of PTX, ESBP-BSANPs-PTX, BSANPs-PTX and BSANPs were added to cells to final drug concentrations ranging from 0.5 ng/mL to 250 ng/mL for RAW264.7 and SK-OV-3; 5 ng/mL to 90 ng/mL for A2780/CP70 and KGN; and 5 ng/mL to 150 ng/mL for HUVEC and ID8. The cell viability of the vehicle (blank) group was considered 100%. The cell viability of each concentration in the ESBP-BSANPs-PTX treatment group was compared with that of the lowest relative concentration in the same group separately, and the statistics are indicated in the graph. The results of the comparison between ESBP-BSANPs-PTX and PTX are shown on the right side of each panel: ^a^ **, 25 ng/mL ESBP-BSANPs vs. 25 ng/mL PTX; ^b^ **, 50 ng/mL ESBP-BSANPs vs. 50 ng/mL PTX; ^c^ *, 1 ng/mL ESBP-BSANPs vs. 1 ng/mL PTX; ^d^ *, 150 ng/mL ESBP-BSANPs vs. 150 ng/mL PTX; ^e^ **, 250 ng/mL ESBP-BSANPs vs. 250 ng/mL PTX; ^f^ **, 10 ng/mL ESBP-BSANPs vs. 10 ng/mL PTX. ns represents *p* > 0.05; ** *p* < 0.01, *** *p* < 0.001 and **** *p* < 0.0001.

The inhibitory effect of ESBP-BSANPs-PTX on all cell lines increased in a dose-dependent trend (Figure 2 and Supplementary Figure S1). ESBP-BSANPs-PTX presented the greatest inhibitory effect at the 72 h time point for each cell line. The 72 h median inhibitory concentrations (IC50) were calculated. Compared to free PTX at the same concentration, ESBP-BSANPs-PTX exhibited a higher cytotoxicity on cell lines HUVEC, RAW264.7, A2780/CP70 and ID8. Compared to BANPs-PTX, ESBP-BSANPs-PTX exhibited a higher cytotoxicity on HUVEC, A2780/CP70 and SK-OV-3.

At the 72 h time point, the ESBP-BSANPs-PTX IC50 and BSANPs-PTX IC50 for A2780/CP70 were 13.77 ng/mL and 30.85 ng/mL; for HUVEC, they were 22.75 ng/mL and 26.15 ng/mL; and those for SK-OV-3 were 12.88 ng/mL and 24.84 ng/mL. RAW264.7 had the ESBP-BSANPs-PTX IC50 of 81.27 ng/mL and BSANPs-PTX IC50 of 226.3 ng/mL. The PTX IC50 for A2780/CP70, HUVEC, SK-OV-3, RAW264.7 and KGN were 21.9189 ng/mL, 27.33 ng/mL, 6.10774 ng/mL, 203.3 ng/mL and 1.42599 ng/mL. ESBP-BSANPs-PTX had higher cytotoxicity for cell lines expressing E-selection (HUVEC and RAW264.7) and cell lines with low CD44 expression (A2780/CP70 and ID8). Those cell lines had good drug uptake results, both of which were higher than free FITC.

The cytotoxicity of BSANPs was not obvious, and its influence on cell survival rate when used as a drug carrier can be considered negligible.

### 3.3. ESBP-BSANPs-PTX Induced Apoptosis and Inhibited Cell Migration in Ovarian Cancer Cells

**Figure 3:**
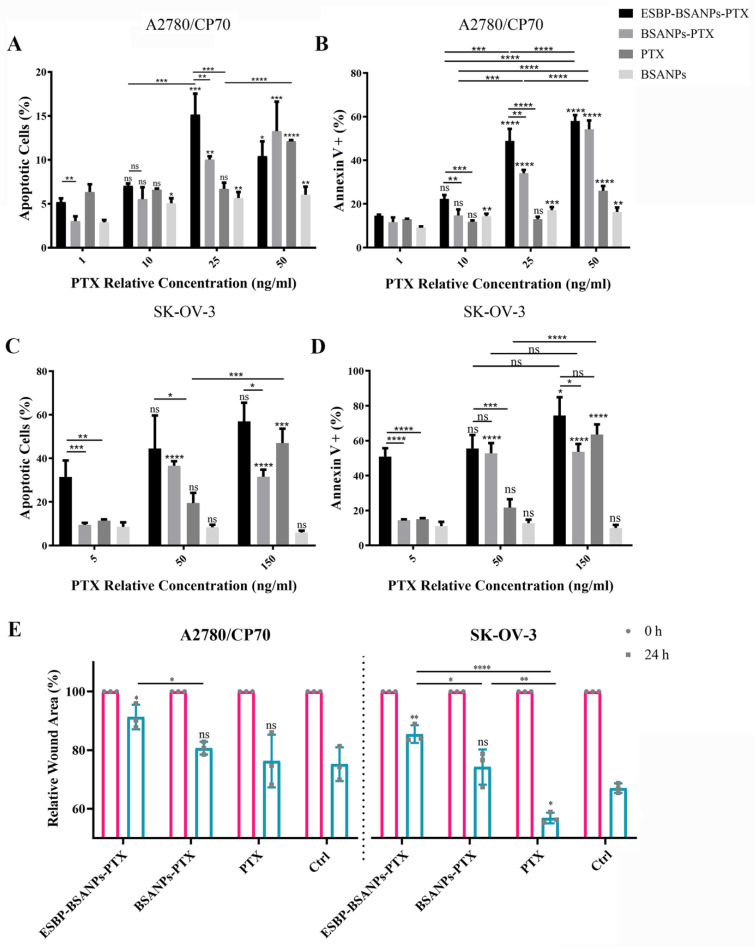
The apoptotic (PI+/Annexin V+) and Annexin V+ (PI+/Annexin V+ and PI-/Annexin V+) cells of (**A**,**B**) SK-OV-3 and (**C**,**D**) A2780/CP70 cell lines after 72 h of drug treatment with serial dilutions (mean ± SD, *n* = 3). The apoptosis and Annexin V+ percentages of each concentration were compared with that of the lowest relative concentration in the same drug treatment group separately, and the statistics are indicated above each error bar. The control groups (cultured in complete medium, 0 ng/mL of drug): untreated SK-OV-3 had an apoptotic result of 8.13 ± 5.97%, Annexin V+ result of 8.86 ± 4.98% and PI-/Annexin V− result of 91.10 ± 4.95%; the untreated A2780/CP70 cells had an apoptotic result of 6.13 ± 1.10%, Annexin V+ result of 11.95 ± 2.07% and PI-/Annexin V− result of 86.78 ± 2.62%. The drug inhibition of cell migration was analyzed using a wound healing assay: the relative wound area (%) of (**E**) A2780/CP70 and SK-OV-3 cells (mean ± SD, *n* = 3) in different treatment groups (relative PTX concentration: 1 ng/mL) is shown. ns represents *p* > 0.05; * *p* < 0.05, ** *p* < 0.01, *** *p* < 0.001 and **** *p* < 0.0001.

To estimate the effect of ESBP-BSANPs-PTX on the apoptosis of ovarian cancer cells, flow cytometry analysis using double staining with annexin V-FITC/PI was performed in A2780/CP70 and SK-OV-3 cells. After being treated with ESBP-BSANPs-PTX for 72 h, and compared with the untreated cells, the apoptotic cells in the treated cells significantly increased in a dose-dependent manner (Figure 3A). When the relative concentrations were 1 ng/mL and 25 ng/mL, the apoptosis of ESBP-BSANPs-PTX in A2780/CP70 was higher than that of BSANPs-PTX (both *p* < 0.01) (Figure 3A). When the relative concentrations were 5 ng/mL and 150 ng/mL, the apoptosis of ESBP-BSANPs-PTX in SK-OV-3 was higher than that of BSANPs-PTX (*p* < 0.001 and *p* < 0.05) (Figure 3C). The percentages of Annexin V+ cells in A2780/CP70 and SK-OV-3 cells increased in a dose-dependent manner (Figure 3B,D).

To estimate the effect of ESBP-BSANPs-PTX on cell migration, wound healing was analyzed in A2780/CP70 and SK-OV-3 cells (Figure 3E). Compared with the blank control (Figure 3E), ESBP-BSANPs-PTX inhibited SK-OV-3 and A2780/CP70 migration (*p* < 0.01 and *p* < 0.05). The inhibition effect of ESBP-BSANPs-PTX was higher than that of BSANPs-PTX (*p* < 0.05) for both cell lines and was higher than that of free PTX (*p* < 0.0001) for SK-OV-3. 

### 3.4. ESBP-BSANPs Sucessfully Targeted Tumor Sites In Vivo

To investigate the in vivo tumor-targeting ability and drug delivery of ESBP-BSANPs, we detected the distribution of ESBP-BSANPs-DID in a mouse subcutaneous tumor model.

**Figure 4:**
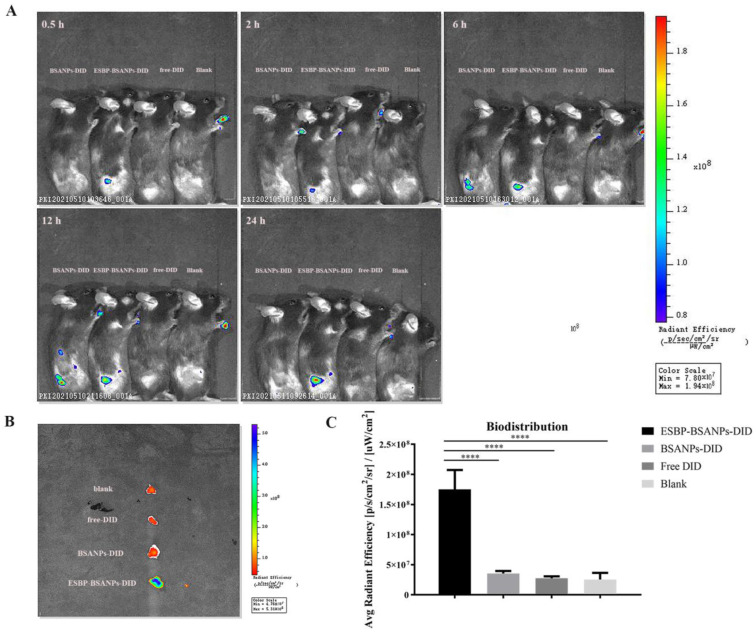
In vivo tumor targeting and drug delivery in mice tumor-bearing model: (**A**) fluorescence distribution in vivo at 0.5 h, 2 h, 6 h, 12 h and 24 h; (**B**,**C**) subcutaneous tumors were removed after 24 h and tumor fluorescence intensity was detected (mean ± SD, *n* = 3). **** *p* < 0.0001.

As shown in Figure 4A, the fluorescence intensity began to increase in the tumor 0.5 h after the administration of ESBP-BSANPs-DID, BSANPs-DID, and free DID, suggesting aggregation in the tumor over time. Figure 4B,C shows the fluorescence imaging results ex vivo after 24 h, which demonstrates that DID was notably delivered and concentrated in the ESBP-BSANPs-DID group tumor site, and was significantly higher than the fluorescence intensity compared to the BSANPs-DID and free DID group tumor sites (*p* < 0.0001). This confirmed that E-selectin was effectively targeted and ESBP-BSANPs could quickly and specifically identify the tumor site. Increased drug accumulation at the tumor site was important for improving drug efficacy.

### 3.5. ESBP-BSANPs-PTX Induced Apoptosis in Ascites and Reduced MDSCs

After the mouse tumor model was established, H&E staining was used to confirm the successful establishment of both abdominal tumor and subcutaneous tumor models before conducting further experiments. Supplementary Figure S2A shows the tumors taken from different groups of mice on the 55th day, which was also the 48th day after the first drug administration. The positive expression of E-selectin in tumor tissue and ascitic cells was investigated via IHC (Supplementary Figure S2B,C).

**Figure 5:**
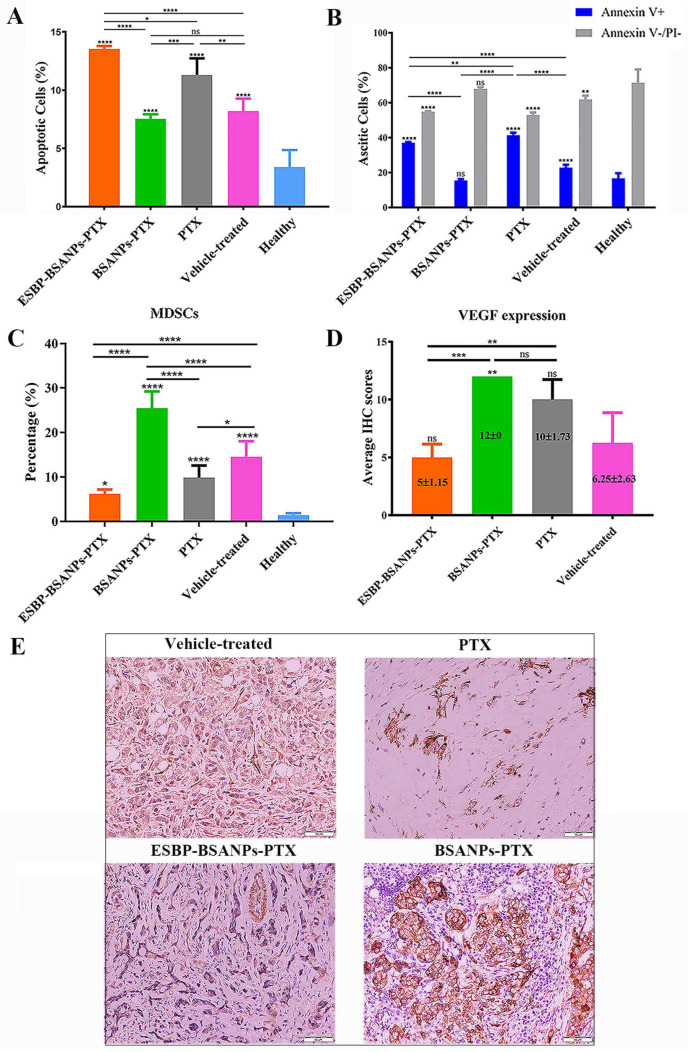
After four rounds of chemotherapy, the (**A**) apoptosis (PI-/Annexin V+) and (**B**) Annexin V+ staining of ascitic/peritoneal washing fluid cells in each treatment group were detected via flow cytometry (mean ± SD, *n* = 5). The apoptosis and Annexin V+ staining of each treatment group was compared with that of the healthy group separately, and the statistics are indicated above each error bar. The PI-/Annexin V− cells in (**B**) indicate the live cells in each treatment group. (**C**) MDSCs in ascitic/peritoneal washing fluid of each treatment group were detected using flow cytometry (mean ± SD, *n* = 5). The MDSCs of each treatment group were compared with that of the healthy group separately, and the statistics are indicated above each error bar. (**D**,**E**) VEGF expression in mouse peritoneal tumor tissue was detected via IHC and photographed (100 times magnification, scale bar: 50 µm). ns represents *p* > 0.05; * *p* < 0.05, ** *p* < 0.01, *** *p* < 0.001 and **** *p* < 0.0001.

In mouse ascites or peritoneal washing fluids (Figure 5A,B), the ESBP-BSANPs-PTX-treated group had more apoptotic cells than the BSANPs-PTX-treated group (*p* < 0.0001), PTX-treated group (*p* < 0.05), the vehicle-treated control group (*p* < 0.0001) and the healthy group (*p* < 0.0001). The ESBP-BSANPs-PTX-treated group had a larger Annexin V+ cell population than the BSANPs-PTX- (*p* < 0.0001) and the vehicle-treated control group (*p* < 0.0001), but a smaller one than that of PTX-treated group (*p* < 0.01). Casepase 3 was positive in all treatment groups and healthy mice (Supplementary Figure S2D).

After four rounds of chemotherapy, the ESBP-BSANPs-PTX-treated group had fewer MDSCs than the BSANPs-PTX-treated group and vehicle-treated control group (both *p* < 0.0001) (Figure 5C). In the BSANPs-PTX-treated group, MDSCs were higher in number than that of PTX-treated group and vehicle-treated control group (both *p* < 0.0001). To further explain the MDSC changes, VEGF expression was detected using IHC (Figure 5D,E). VEGF decreased in the ESBP-BSANPs-PTX-treated group, and its expression was lower than in the BSANPs-PTX-treated group and PTX-treated group (*p* < 0.001, *p* < 0.01). 

### 3.6. ESBP-BSANPs-PTX Inhibited Tumor Growth In Vivo and Prolonged Survival Time

**Figure 6:**
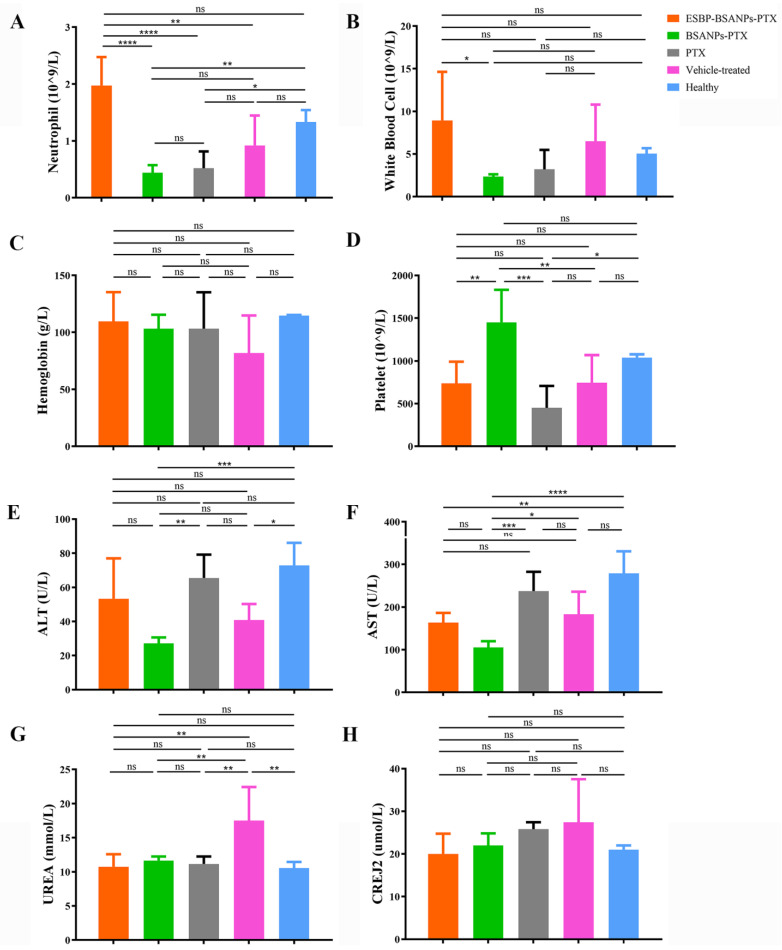
Blood test results after four cycles of treatment: (**A**) neutrophil number (Gran#), (**B**) white blood cell (WBC), (**C**) hemoglobin (HGB), (**D**) platelets (PLT), (**E**) alanine aminotransferase (ALT), (**F**) aspartate aminotransferase (AST), (**G**) urea (UREA) and (**H**) creatinine (CREJ2) (mean ± SD, *n* = 5). ns represents *p* > 0.05; * *p* < 0.05, ** *p* < 0.01, *** *p* < 0.001 and **** *p* < 0.0001.

After four cycles of chemotherapy, the blood neutrophil number (Figure 6A) of ESBP-BSANPs-PTX-treated mice had no significant difference from healthy mice (*p* > 0.05), but was higher than those of vehicle-treated control group (*p* < 0.01). The number of neutrophils in PTX-treated mice and BSANPs-PTX-treated mice was lower than that of healthy mice (*p* < 0.05, *p* < 0.01).

The WBC, HGB and PLT results in each group were within normal ranges (Figure 6B–D). PLT in the ESBP-BSANPs-PTX-treated group (735.6 ± 256.5 × 10^9^/L) was less than that of the BSANPs-PTX-treated group (1451.0 ± 380.6 × 10^9^/L) (*p* < 0.01), but there was no significant difference compared with the PTX-treated (450.6 ± 257.2 × 10^9^/L), vehicle-treated control (743.8 ± 324.1 × 10^9^/L) and healthy groups (1039.7 ± 37.9 × 10^9^/L) (*p* > 0.05). 

Liver and kidney functions were examined by comparing the ALT, AST, UREA and CREJ2 results of the experimental groups with the healthy group (Figure 6E–H). Each of the ESBP-BSANPs-PTX-, BSANPs-PTX-, and PTX-treated groups had normal liver and kidney function, while the ESBP-BSANPs-PTX group had a lower AST result (*p* < 0.01), and the BSANPs-PTX group had lower ALT and AST results (*p* < 0.001 and *p* < 0.0001) than that of healthy mice. However, the vehicle-treated control group showed an abnormality in kidney function with higher UREA (*p* < 0.01).

**Figure 7:**
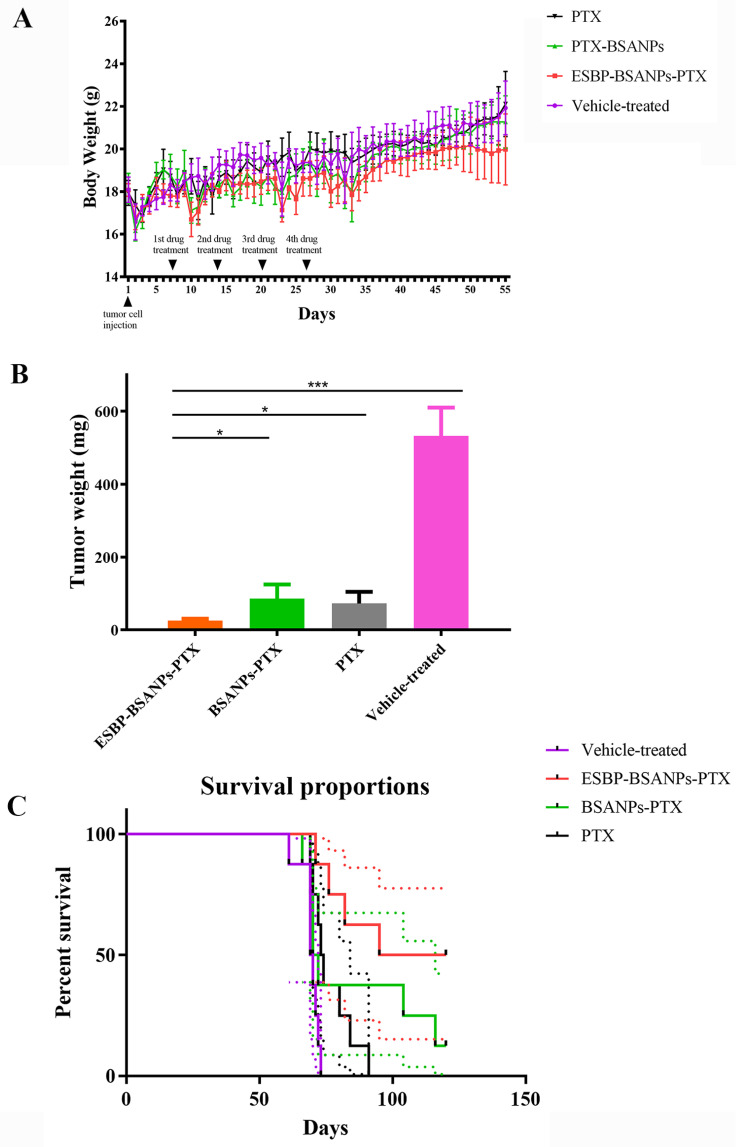
After four cycles of treatment: (**A**) body-weight time curve in each group of mice for 55 days (mean ± SD, *n* = 8). (**B**) Tumor weight in each group of mice after finishing four cycles of treatment was calculated (mean ± SD, *n* = 5). (**C**) 120-day survival curves of mice in different treatment groups (ESBP-BSANPs-PTX-, BSANPs-PTX-, PTX-treated and vehicle-treated control group) (*n* = 8). * *p* < 0.05 and *** *p* < 0.001.

After tumor cell injection, the body weights of every group had no significant difference (*p* > 0.05) (Figure 7A). The tumors were weighed after mice in each group were sacrificed (Figure 7B). Tumor weight for the ESBP-BSANPs-PTX-, BSANPs-PTX-, and PTX-treated groups decreased after treatment, while the ESBP-BSANPs-PTX-treated group had lighter tumors than other groups (*p* < 0.05, *p* < 0.05 and *p* < 0.001).

The median survival time was calculated using survival curves (Figure 7C): the ESBP-BSANPs-PTX-treated group’s was 107.5 days, the vehicle-treated control group’s was 69.5 days, the BSANPs-PTX group’s was 71 days, and the PTX-treated group’s was 73.5 days. The median survival time of ESBP-BSANPs-PTX was the longest, indicating an extended survival. The survival time of mice in the ESBP-BSANPs-PTX administration group was significantly longer than that in PTX treatment group and control group (*p* < 0.01, *p* < 0.001).

## 4. Discussion

At present, the main treatments for ovarian cancer include maximal surgical cytoreduction and chemotherapy [2,3]. PTX is one of the first-line chemotherapeutics. However, PTX has severe side effects on healthy cells [6,7,8,9], and has limitations such as drug resistance. Nab-paclitaxel uses albumin associated with paclitaxel as a carrier for drug internalization in tumor cells [24,25], and its uptake is facilitated by the interaction of albumin with the cell surface receptor gp60 expressed on endothelial cells [26,27]. However, nab-paclitaxel is passively accumulated in cells and cannot selectively deliver PTX to a certain site. Modification of structures can further improve the efficacy and compatibility of nanoparticles. Conjugating with specific ligands allows nanoparticles to achieve targeted delivery to a tumor site, thus enhancing therapeutic efficacy.

E-selectin was rarely studied in ovarian cancer treatment, and studies are mostly about its level changes as one of the vasoactive and inflammatory mediators [28,29]. In this study, we developed ESBP-BSANPs-PTX to treat ovarian cancer. E-selectin-binding peptide (ESBP) was selected as the high-affinity ligand to deliver drugs to inflammatory vascular endothelial cells in a tumor site. Both in vivo and in vitro targeting results showed that ESBP-modified BSANPs (ESBP-BSANPs) improved the biocompatibility of PTX and selective PTX delivery to ovarian cancer cells and inflammatory vascular endothelial cells (HUVEC activated with LPS). ESBP-BSANPs showed its selective drug delivery. The increased uptake of ESBP-BSANPs-FITC by LPS-activated HUVEC proved that cellular uptake was related to the specific binding between ESBP and E-selectin on the cell surface and this happened more in inflammatory sites. That means that the E-selectin produced by cells has a specific interaction with ESBP, which promoted the binding of nanoparticles to cells.

Remarkably, all six cell lines have a higher uptake of ESBP-BSANPs-FITC than free FITC; thus, the targeting of ovarian tumor cells and inflammatory vascular endothelial cells guarantees a higher cytotoxicity for cells in tumor sites and a lower toxicity for healthy cells. In this study, BSANPs had a high IC50 for each cell line, also demonstrating that the nanoparticle treatment did not significantly impact normal cell growth and had good cellular compatibility. 

E-selectin promotes cancer metastatic behavior [30] and facilitates the adhesion of circulating tumor cells, leading to the preferential homing [31] and retention of metastatic cancer cells [32]. CD44, one of the E-selectin ligands expressed on migrating cancer cells [33,34], is known to interact with the soluble protein E-selectin in these processes [35,36]. According to our previous research, both E-selectin and CD44 are highly expressed in ovarian cancer tissue. In this paper, we reported the higher uptake of ESBP-BSANPs-FITC in ovarian cancer cells with low CD44 expression (A2780/CP70 and ID8) than that of BSANPs-FITC, and the lower IC50 of ESBP-BSANPs-PTX in these cell lines than that of free PTX. CD44 was proven to be associated with drug resistance and the tumor metastasis of ovarian cancer in studies [37,38,39,40,41]. CD44 expression showed its influence on the efficacy of ESBP-BSANPs, but ESBP-BSANPs-PTX still demonstrated its inhibition of cell migration in both the CD44 low-expression cell lines, A2780/CP70, and CD44 high-expression cell line SK-OV-3. The diameter of ESBP-BSANPs-PTX is 172.57 ± 14.63 nm, so it has greater steric hindrance than the other substances in interstitial cell fluid. This steric hindrance may increase the macromolecular crowding effect of the intercellular cell fluid [42,43,44,45] when cells are identified by ESBP and are bound. This wrapping of cancer cells can prevent the membrane from moving. Therefore, it may reduce the amoeba movement of tumor cells and reduce metastasis.

MDSCs in mice have two distinct subtypes: CD11bLy-6GLy-6C^++high^ and CD11bLy-6GLy-6C^++low^ [46]; we detected both subtypes using flow cytometry and found an apparent reduction in the ESBP-BSANPs-PTX-treated group, showing a lower drug resistance and better drug effect in vivo. MDSCs are a population of immature myeloid cells that accumulate in patients with cancer [32] and have immunosuppressive properties [47]. MDSCs have been shown to play pleiotropic roles in cancer progression by promoting tumor cell proliferation, metastatic growth and drug resistance through immunosuppression and inflammation [48]. Reducing MDSCs in a tumor microenvironment can be a promising and feasible strategy to attenuate tumor progression. Previous research showed that VEGFA produced by ovarian cancer cells stimulates MDSC migration and differentiation through VEGFR1 expression in MDSCs [49]. In the tumor-bearing mice model, we noticed a downregulated VEGF in the ESBP-BSANPs-PTX-treated group compared with the free PTX-treated group. VEGF expression can be downregulated by PTX [50]; the lower VEGF level in the ESBP-BSANPs-PTX-treated group could potentially reduce the number of MDSCs in ascites. 

According to the better blood test results, ESBP-BSANPs-PTX had higher number of neutrophils and is proven to have the ability to reduce side effects such as neutropenia. Neutropenia is known to be the dose-limiting toxicity of nab-paclitaxel and PTX [51], and low baseline platelet levels are considered to be an associated risk factor [52]. Previous studies have also reported a similar association for other chemotherapeutic regimens [53]. As mentioned in the result, the BSANPs-PTX- and PTX-treated groups had lower neutrophil counts than healthy mice, while the platelet level for the BSANPs-PTX group was higher than in healthy mice and that of the PTX group was lower than the healthy group. Studies showed the neutrophils and platelet levels can be related to outcome and recovery following chemotherapy [52,54], and thus, it can be predicted that ESBP-BSANPs-PTX treatment may lead to better hematological recovery and outcome than BSANPs-PTX and PTX treatments. This prediction is supported by the prolonged survival time of the ESBP-BSANPs-PTX-treated mice.

## 5. Conclusions

In conclusion, our present results demonstrated the targetability and anti-tumor effect of ESBP-BSANPs-PTX. The ESBP-BSANPs-PTX that we investigated could use ESBP to recognize E-selectin and actively target tumors, so allowing a drug delivery system to actively recognize the cells and selectively deliver drugs to a tumor site. ESBP-BSANPs-PTX enhanced the efficacy of PTX, exhibiting higher cytotoxicity and anti-proliferation ability in ovarian cancer cells compared to free PTX. Moreover, ESBP-BSANPs-PTX treatment induced apoptosis and inhibited cell migration. ESBP-BSANPs-PTX had a lower in vivo toxicity than free PTX and a higher drug-delivery ability to mouse tumor sites. Furthermore, the reduction in the number of MDSCs in the ESBP-BSANPs-PTX group indicated lower drug resistance in this treatment group. Our present results provided evidence that ESBP-BSANPs-PTX may be a potential anti-cancer agent in ovarian cancer.

However, based on E-selectin’s expression at inflammation and infection sites, the selective and specific drug delivery to cancer tissue, but not to non-cancer inflammation sites, remains to be further proven and addressed in future research.

## Data Availability

The data presented in this study are available on request from the corresponding author.

## Supplementary Materials

The following supporting information can be downloaded at: https://www.mdpi.com/article/10.3390/cancers15072136/s1, Figure S1: Cell viability of ovarian cancer cell lines. (A) A2780/CP70, (B) ID8, (C) SK-OV-3) and (D) HU-VEC, (E) RAW264.7, (F) KGN cells for 24h and 48h time points was analyzed by microplate read-er (mean ± SD, n = 3). Serial dilutions of PTX, ESBP-BSANPs-PTX, BSANPs-PTX, and BSANPs were added to cells to final drug concentrations ranging from 0.5 ng/mL to 250 ng/mL for RAW264.7 and SK-OV-3; 5 ng/mL to 90 ng/mL for A2780/CP70 and KGN; 5 ng/mL to 150 ng/mL for HUVEC and ID8. The cell viability of vehicle (blank) group was considered 100%. The cell viability of each concentration in ESBP-BSANPs-PTX treatment group was compared with that of the lowest relative concentration in the same group separately, and the statistics were indicated in the graph; Figure S2: (A) H&E staining showed that the gray-white nodules taken from the model mice were cancerous nodules, and adenoid differentiated tumor cells could be seen (100 times magnification, scale bar: 100 µm). (B) IHC detection of diffuse positive (+++) expression of E-selectin in mouse peritoneal tumor tissue and (C) positive (+) expression of CD62E in the ascitic cells of the mouse model (100 times magnification, scale bar: 100 µm). (D) IHC results of Casepase 3 detected in ascitic/peritoneal washing fluid of each group were all positive (+) (200 times magnification, scale bar: 50 µm).
