# Supplementary material for: Inflammation Control and Tumor Growth Inhibition of Ovarian Cancer by Targeting Adhesion Molecules of E-Selectin
Source: Cancers (Basel). 2023 Apr 4;15(7):2136. doi: 10.3390/cancers15072136 (PMC10093113; doi:10.3390/cancers15072136)
Supplement: Supplementary file 1 [file cancers-15-02136-s001.zip › cancers-2236006-supplementary.pdf]

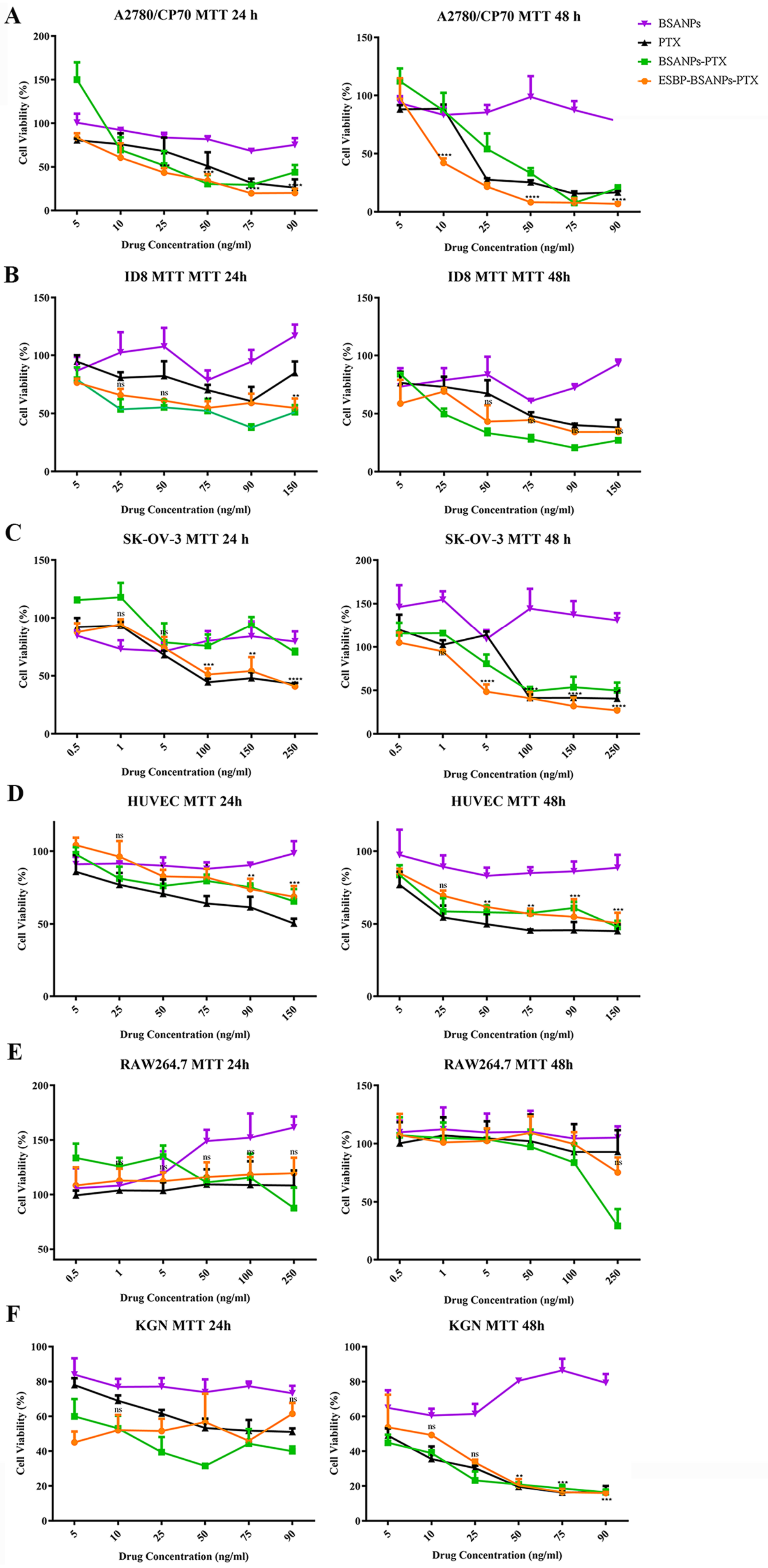
A
A2780/CP70 MTT 24 h
A2780/CP70 MTT 48 h
BSANPs
PTX
BSANPs-PTX
ESBP-BSANPs-PTX
B
ID8 MTT MTT 24h
ID8 MTT MTT 48h
C
SK-OV-3 MTT 24 h
SK-OV-3 MTT 48 h
D
HUVEC MTT 24h
HUVEC MTT 48h
E
RAW264.7 MTT 24h
RAW264.7 MTT 48h
F
KGN MTT 24h
KGN MTT 48h
Cell Viability (%)
Drug Concentration (ng/ml)

Figure S1. Cell viability of ovarian cancer cell lines. (**A**) A2780/CP70, (**B**) ID8, (**C**) SK-OV-3) and (**D**) HUVEC, (**E**) RAW264.7, (**F**) KGN cells for 24h and 48h time points was analyzed by microplate reader (mean ± SD, n=3). Serial dilutions of PTX, ESBP-BSANPs-PTX, BSANPs-PTX, and BSANPs were added to cells to final drug concentrations ranging from 0.5ng/ml to 250ng/ml for RAW264.7 and SK-OV-3; 5ng/ml to 90ng/ml for A2780/CP70 and KGN; 5ng/ml to 150ng/ml for HUVEC and ID8. The cell viability of vehicle (blank) group was considered 100%. The cell viability of each concentration in ESBP-BSANPs-PTX treatment group was compared with that of the lowest relative concentration in the same group separately, and the statistics were indicated in the graph.

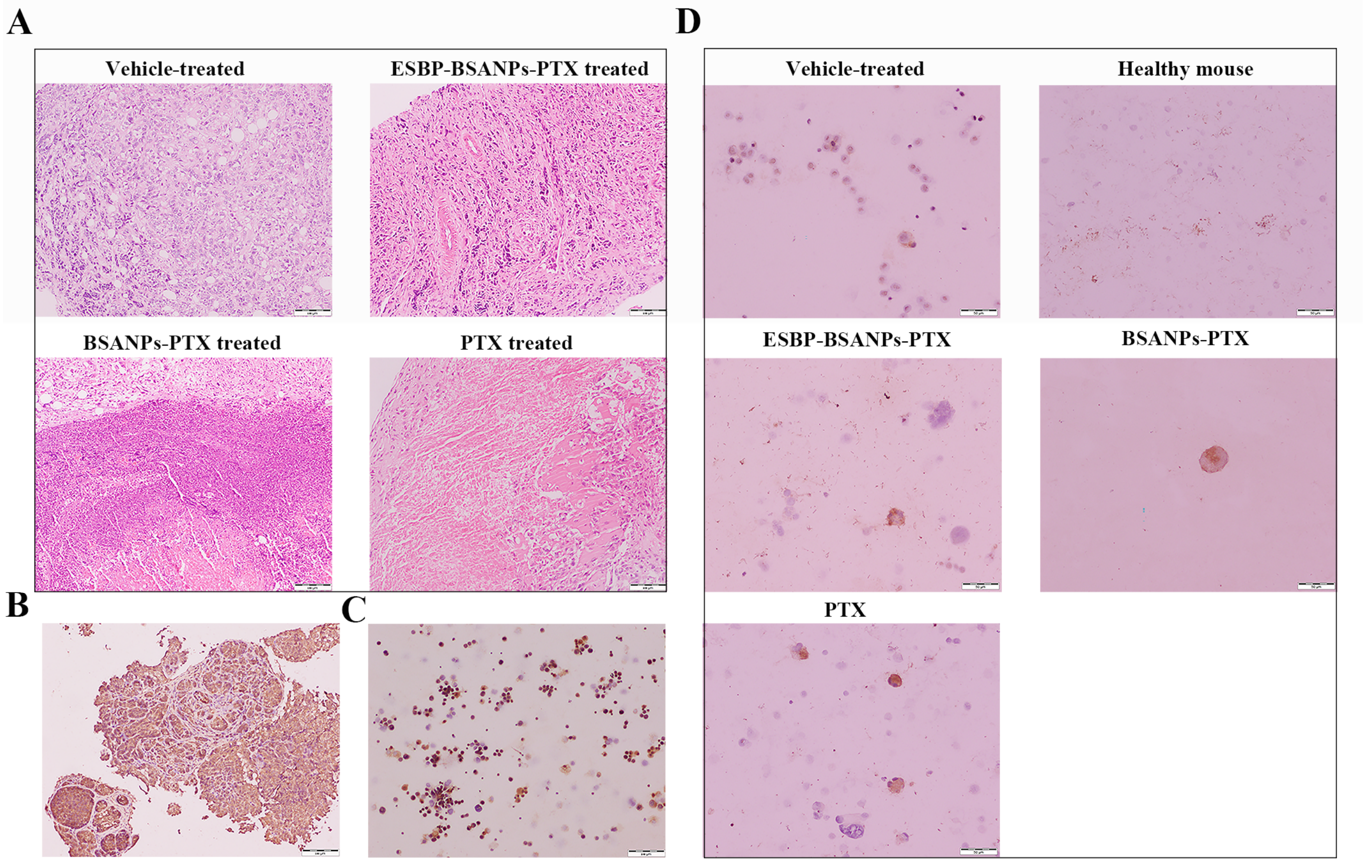

Figure S2. (**A**) H&E staining showed that the gray-white nodules taken from the model mice were cancerous nodules, and adenoid differentiated tumor cells could be seen (100 times magnification, scale bar: 100 μm). (**B**) IHC detection of diffuse positive (+++) expression of E-selectin in mouse peritoneal tumor tissue and (**C**) positive (+) expression of CD62E in the ascitic cells of the mouse model (100 times magnification, scale bar: 100 μm). (**D**) IHC results of Casepase 3 detected in ascitic/peritoneal washing fluid of each group were all positive (+) (200 times magnification, scale bar: 50 μm).
